# Blood Vessel Detection Algorithm for Tissue Engineering and Quantitative Histology

**DOI:** 10.1007/s10439-022-02923-2

**Published:** 2022-02-16

**Authors:** A. Adamo, A. Bruno, G. Menallo, M. G. Francipane, M. Fazzari, R. Pirrone, E. Ardizzone, W. R. Wagner, A. D’Amore

**Affiliations:** 1 Department of Health Promotion, Mother and Child Care, Internal Medicine and Medical Specialties, University of Palermo, 90100 Palermo, Italy; 2 McGowan Institute for Regenerative Medicine, University of Pittsburgh, Pittsburgh, PA 15219 USA; 3 Fondazione Ri.MED, 90133 Palermo, Italy; 4 Department of Computing and Informatics in the Faculty of Science and Technology, Bournemouth University, Poole, BH12 5BB UK; 5 Department of Biomedical Engineering, Worcester Polytechnic Institute, Worcester, MA 01605 USA; 6 Department of Pathology, University of Pittsburgh, Pittsburgh, PA 15206 USA; 7 Department of Pharmacology & Chemical Biology, University of Pittsburgh, Pittsburgh, PA 15261 USA; 8 Department of Industrial and Digital Innovation, University of Palermo, 90100 Palermo, Italy; 9 Department of Surgery, University of Pittsburgh, Pittsburgh, PA 15219 USA; 10 Department of Surgery and Bioengineering, McGowan Institute for Regenerative Medicine, University of Pittsburgh, 450 Technology Drive, Pittsburgh, PA 15219 USA

**Keywords:** Angiogenesis, Vascularization, Blood vessel formation, Quantitative histology, Quantitative immunohistochemistry, Biomaterials host response, Blood vessel morphology, Automated image analysis

## Abstract

Immunohistochemistry for vascular network analysis plays a fundamental role in basic science, translational research and clinical practice. However, identifying vascularization in histological tissue images is time consuming and markedly depends on the operator’s experience. In this study, we present “blood vessel detection—BVD”, an automatic algorithm for quantitative analysis of blood vessels in immunohistochemical images. BVD is based on extraction and analysis of low-level image features and spatial filtering techniques, which do not require a training phase. BVD algorithm performance was comparatively evaluated on histological sections from three different *in vivo* experiments. Collectively, 173 independent images were analyzed, and the algorithm's results were compared to those obtained by human operators. The developed BVD algorithm proved to be a robust and versatile tool, being able to quantify number, area, and spatial distribution of blood vessels within all three considered histologic datasets. BVD is provided as an open-source application working on different operating systems. BVD is supported by a user-friendly graphical interface designed to facilitate large-scale analysis.

**Supplementary Information:**

The online version contains supplementary material available at 10.1007/s10439-022-02923-2.

## Introduction

The evaluation of angiogenesis and neovascularization has a crucial role in numerous pathological conditions, including lower-extremity peripheral artery disease^20^ that is associated with insufficient blood supply, or chronic inflammatory diseases^6^ and cancer,^13,14^ where Blood Vessel (BV) spatial density and morphology are used to monitor tumor progression.^2,4^ BV quantification is relevant for tissue engineering applications as well,^7,8^ as the development of a functional vascular network constitutes an essential element in endogenous tissue growth,^34^ healing, tissue repair and *de novo* tissue formation.^4,36^

Immunohistochemistry (IHC) is a powerful technique to identify specific structures in cells of a tissue section. Immunofluorescence (IF) is a specific example of IHC. In both cases, IHC and IF, a primary antibody is used against a target molecule within the section, and a marked secondary antibody is used to visualize the molecule distribution. IHC target molecules can be visualized with visible light using chromogenic dyes^39^; on the other hand, IF uses fluorescence probes, and the signal is observed using a conventional epifluorescence microscope of confocal microscopy.^22^ Owing to their versatility, these techniques are widely accepted methods for quantification and classification of BVs in clinical and basic science, allowing easier visualization of the structures of interest that are positively marked in contrast to an unmarked background.^33^ In particular, IF imaging is often used in tissue engineering context due to its specificity. However, polymeric biomaterials are often affected by the autofluorescence or other interfering optical phenomena^23^

From the, somehow simplified, prospective of developing an immunostaining strategy, BVs consist of an inner, thin layer of Endothelial Cells (EC) surrounded by smooth muscle cells or pericytes. Staining techniques for BVs are commonly based on labeling the endothelium with antibodies binding specifically to EC antigens such as Willebrand factor, CD31, and CD34, and a second antibody binding to Smooth Muscle Alpha Actin (αSMA) in smooth muscle cells.^1^ While antibody specificity and sensitivity have reached maturation, technical aspects of tissue processing and IHC can alter the histological section appearance or be a source of experimental variability. Tissue heterogeneity, inadequate section thickness, sample folds or wrinkles, suboptimal amplification or blocking protocols, insufficient concentration, and reactivity of the staining reagents, among others, can affect the quality and the accuracy of analysis.^2,4^ Each of these steps is crucial in the immunologic reactions, especially when in the presence of implanted biomaterials. When the protocol is inappropriate, the antibodies can bind to unrelated antigens in the tissue/biomaterial section, resulting in high background, unspecific staining, or masked antigenic sites. Despite the efforts to standardize IHC staining protocols^27^ for BV detection in histological sections, due to the large number of manual steps involved absolute standardization remains unattainable. The quantitative assessment of vascularization not only requires effective staining protocols, but also automatic, rapid and unbiased methods of analysis.

The current practice for the histological analysis is still based on human operators,^26,37^ an activity that is time consuming, prone to operator subjectivity^24,30^ and inter/intra observer variability.^18,26^ These limitations can be all addressed by the development of methods for digital image analysis. Over the last years, the interest in computer-based methods for BV quantification has increased, yet accessible, ready-to-use, and user-friendly software for quantitative analysis of vascularization is poorly developed. Multiple strategies have been explored, predominantly in the context of tumor progression, to address angiogenesis quantification, including counting vessels^3,5,17,25,38^ and extracting morphological features such as BV surface and perimeter.^5,9,12,25,40^ However, most of these methods suffer limitations, especially in the tissue engineering field, as their applicability and the experimental dataset adopted for the validation were limited to pathological tissue. Furthermore, these^3,5,9,12,17,25,38,40^ algorithms are not open source, do not have a stand-alone app, and require interaction with an expert user or image pre-processing. Only a few of them offer a complete morphometrical and topological characterization of the sections.^12,31,32^ Conversely, morphometrical measurements are all relevant parameters for the analysis of the histological section, not only for diagnostic applications but also for bioengineering, regenerative medicine, and pre-clinical models.

In the present study, an automatic and ready-to-use morphological tool for quantitative analysis of BV is presented. The developed Blood Vessel Detection (BVD) software allows: (I) to quantify the number and size of BVs; (II) to identify their position within the specimen; (III) to measure the vascular area fraction for pixels that are positive to individual or multiple staining; and (IV) to produce a topological representation of the vascularization within a Region of Interest (ROI). The method was tested and validated using data from three independent *in vivo* experiments that specifically focused on the characterization of angiogenesis.^7,15,35^ Three different tissue types were adopted including: cardiac muscle, abdominal wall, and metanephric kidney engrafted in the omentum*.* IHC staining of vascular structures in the three *in vivo* studies was based on the labeling of the EC marker CD31, and/or αSMA. Efficiency and efficacy of BVD algorithm was assessed with head-to-head comparison of human vs. algorithm detected vessel number, as well as with known metrics for algorithm performance such as *precision*, *recall* and *F-measure*.^28,29^ False positive and negative, were also comparatively studied and discussed.

## Materials and Methods

### Experimental Dataset Utilized to Validate the Algorithm

#### Dataset 1: Drug-Controlled Release in Rat Abdominal Wall Defect Model

The first set of data analyzed offers a common example of drug-controlled release. A rat abdominal wall defect model was treated with an elastomeric patch loaded with micro-particles able to release an angiogenic factor.^7^ Adult female Sprague-Dawley rats (Harlan Sprague Dawley, Inc., Indianapolis, IN) were used to implement a partial abdominal wall defect model. A poly(ester urethane) urea (PCUU) electrospun-scaffold, integrated with porcine Extracellular Matrix (ECM) gel and loaded with poly(lactic-co-glycolic acid) (PLGA) microparticles, was implanted as an abdominal wall patch. The PLGA microparticles controlled released Nitro-oleic acid (NO_2_-OA). NO_2_-OA is an electrophilic fatty acid nitro-alkene derivative which, under hypoxic conditions, induces angiogenesis. In addition to the scaffold designed for the controlled release (*scaffold + ECM + NO*_*2*_*-OA*), two controlled groups were considered: a polymeric scaffold integrated with ECM but not carrying the PLGA particles for the drug release (*scaffold + ECM*), and a simple polymeric scaffold not loaded with particles nor integrated with ECM gel (*scaffold*). Histological samples were harvested and preprocessed with fixation on 4% phosphate-buffered paraformaldehyde solution (4 h), followed by immersion in 30% sucrose solution (> 48 h), embedding into OCT compound (Tissue-Tek, Torrance,CA) and finally sectioned with a 10µm step size using a microtome. For the IHC staining, abdominal wall sections were blocked for 2 hours at room temperature with 10% goat serum in 0.2% Triton-PBS solution. Briefly, slides were incubated with mouse primary antibody against CD31 1:100 (Ab64543, Abcam) and rabbit primary antibody against Alpha-SMA 1:100 (Ab5694, Abcam). Anti-rabbit Alexa Fluor® 594 1:1000 (A21207, Invitrogen), biotinylated anti-mouse 1:200 (BA-2001, Vector Laboratories) and Streptavidin-Alexa Fluor® 488 conjugate 1:150 (S32354, Invitrogen) were utilized as secondary antibodies, 4′,6-diamidino-2-phenylindole (DAPI) (DAPI H-1200; Vectashield) was utilized for nuclear staining. Multispectral epifluorescent images were acquired using a Nikon Eclipse 6600 Microscope (Nikon Corporation) with spectral unmixing to remove autofluorescence performed using Nuance 3.0.2 software (Caliper Life Science Inc.).

#### Dataset 2: Tissue Engineered Biohybrid Scaffold in a Rat Infarction Model

The second study provides an example of how the BVD algorithm can be applied to dataset that are commonly generated when a biomaterial is implanted to induce endogenous tissue growth and constructive remodeling.^35^ Adult female Lewis rats (Harlan Sprague Dawley, Inc., Indianapolis, IN) were infarcted by left anterior descending artery ligation as described in.^8^ After 5 days, animals were randomized in three groups: *control*, *Losartan* and *scaffold*. Losartan is an angiotensin II receptor blocker, clinical and experimental data indicate that Losartan is able to induce the expression and the secretion of vascular endothelial growth factors and trigger angiogenesis.^42^ Animals were treated daily receiving oral Losartan (15 mg/kg) until the end of the protocol. After 8 weeks following the infarction, all the animals underwent a second surgery. For the animals in *scaffold group*, a cardiac patch scaffold was epicardially placed and sutured over the infarcted area. The animals in *control* (infarcted and not treated with Losartan) and *Losartan* groups did not receive patch implantation. A poly(ester carbonate urethane)urea (PECUU) electrospun-scaffold enriched with porcine ECM gel layer was used as cardiac patch. In particular, the incorporation of an ECM gel component within a polymeric fibrillar scaffold has been shown to induce angiogenesis and promote constructive remodeling.^41^

After 8 weeks following the treatment surgery, animals were sacrificed and the hearts were harvested. Histological samples were fixed in 4% phosphate-buffered paraformaldehyde solution (4 h), followed by immersion in 30% sucrose solution over-night and finally embedded in OCT compound and sectioned with a 10µm step size using a microtome. For the IHC staining cardiac tissue sections were incubated for 20 minutes at 70°C in antigen retrieval Histo VT One® (Nacalai Tesque, Kyoto, Japan) diluted 10 times in deionized water. Sections were blocked for 3 hours at room temperature with 2% BSA and 10% goat serum in 0.2% Triton-PBS solution. To identify BV, sections were incubated with mouse primary antibody against CD31 1:100 (Ab64543, Abcam) and rabbit primary antibody against Alpha-SMA 1:200 (Ab5694, Abcam). As previously done in the abdominal wall study, anti-rabbit Alexa Fluor® 594 1:1000 (A21207, Invitrogen), biotinylated anti-mouse 1:200 (BA-2001, Vector Laboratories) and Streptavidin-Alexa Fluor® 488 conjugate 1:150 (S32354, Invitrogen) were utilized as secondary antibodies, 4′,6-diamidino-2-phenylindole (DAPI) (DAPI H-1200; Vectashield) was utilized for nuclear staining. Multispectral epifluorescent images were acquired using a Nikon Eclipse 6600 Microscope with spectral unmixing to remove autofluorescence performed using Nuance 3.0.2 software.

#### Dataset 3: Kidney Tissue Organogenesis in the Omentum

The third case provides an example of how the BVD algorithm can be applied to ectopic tissue organogenesis. Metanephric (E14.5) kidneys were retrieved from timed-pregnant C57BL/6J mice as previously described^16^ and transplanted in the omentum of recipient C57BL/6J mice.^15^ After two weeks from the engraftment, the kidneys were retrieved from the mice and prepared for histological examination. Histological samples were fixed in 4% paraformaldehyde, infiltrated in 30% sucrose solution, embedded in OCT compound, and finally sectioned into 4 µm-thick sections for IF analyses. Sections were permeabilized with cold 0.1% Triton X-100 for 10 minutes and nonspecific antibody binding was later prevented by incubating sections in 3% bovine serum albumin solution for 30 minutes. Sections were therefore incubated with anti-CD31 antibody (1:50, ab28364, Abcam) 1 h at room temperature. Alexa Fluor 594 (1:350, A-11012, Thermo Fisher) was used as secondary antibody for 20 minutes. Images were obtained with an Olympus IX71 (Olympus Corporation) inverted fluorescence microscope.

### Image Preparation and Analysis Performed by Human Operators

Images of datasets 1, 2, and 3 were obtained using a Nikon Eclipse 6600 Microscope and an Olympus IX71. Fluorescent probes, including DAPI, FITC (fluorescein isothiocyanate), and TRITC (tetramethylrhodamine isothiocyanate), were detected using an excitation filter with narrow bandpass windows in the violet (385 to 400 nanometers), blue (475 to 490 nanometers), and green (545 to 565 nanometers) spectral regions.

Twelve independent explants from dataset 1^7^ were analyzed: N=3 from *scaffold* group, N=4 from *scaffold + ECM* group and N=5 from *scaffold + ECM + NO*_*2*_*-OA* group. For each explants, 8 random images were collected, this corresponded to a total of 96 analyses. Each extracted image covered a field of view of 0.091 mm^2^, which was equivalent to 697x520 pixels. Images were saved in JPEG format. Three rat heart explants of each group from dataset 2^35^ were examined including: *control*, *scaffold* and *Losartan* groups, for a total of 9 independent specimens and 45 images (5 images/sample). Each image covered a field of view of 0.185 mm^2^ which was equivalent to 994x742 pixels. Images were saved in JPEG format. For both dataset 1 and dataset 2, mature BVs and capillaries were manually detected by one human operator for each study. The operator was blinded to the treatment groups and not involved in the projects. Finally, for the third dataset,^15^ 32 independent glomeruli, in five different images, were analyzed: N=5 from *Image 1*, N=6 from *Image 2*, N=7 from *Image 3*, N=8 from *Image 4* and N=6 from *Image 5*. The field of view for each image acquisition was 0.46 mm^2^, which is equivalent to 1036x1024 pixels. The fluorescence intensity for CD31 positive pixels was measured for each of the glomeruli within the images by selecting regions of interest around the Bowman’s capsule containing the glomerular capillaries. Intensity values were measured processing images in TIFF format *via* ImageJ (developed at NIH), as previously described.^19^ Glomeruli from 5 sections/mouse were considered. The corrected total glomerular fluorescence (CTGF) was calculated using Eq.1^15,19^:

1 $${\text{CTGF}} = {\text{Integrated}}\;{\text{Density}} - \left( {{\text{Area}}\;{\text{of}}\;{\text{selected}}\;{\text{glomerulus}} \times {\text{Mean}}\;{\text{fluorescence}}\;{\text{of}}\;{\text{background}}} \right)$$

Collectively, the algorithm was validated and assessed by utilizing 173 independent images that were previously^7,15^ analyzed by human operators and obtained from three different tissue types.

### Blood Vessel Detection Algorithm Structure

**Figure 1:**
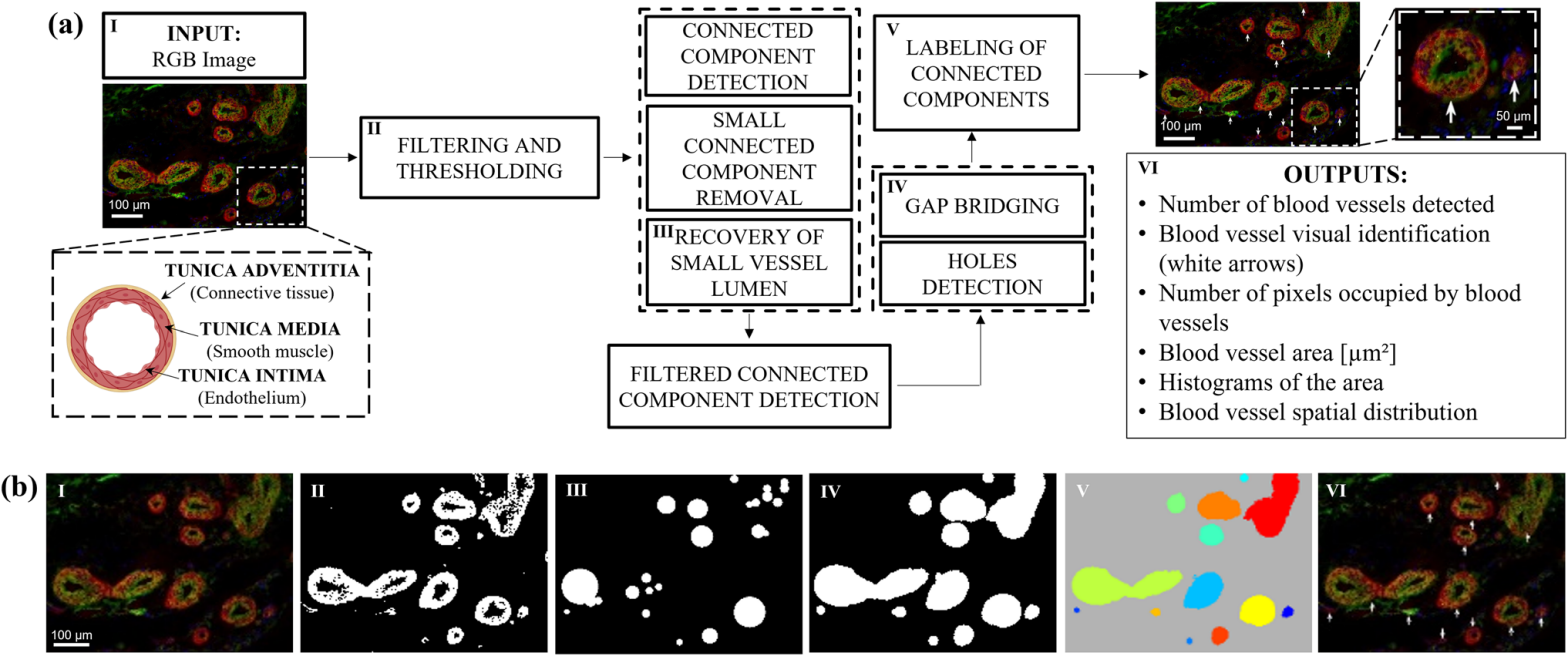
Workflow for blood vessel detection - BVD algorithm. (a) General block diagram of BVD algorithm workflow and its graphical illustration. (b) Representative rat cardiac tissue section co-stained with CD31 + αSMA, scale bar is equal to 100µm, different stages of the processing are illustrated in images (I–VI). (I) Starting RGB input image; (II) image equalized by 3 by 3 averaging spatial filtering and local thresholding; (III–IV) detection of connected components; (V–VI) labeling and extraction of morphological descriptors for the BVs, their location is highlighted with white arrows. The outputs of the analysis (VI) include: number, area (µm^2^ and pixels), spatial distribution, vascular area, and diameter histograms.

The BVD algorithm and its Graphical User Inteface (GUI) (Supplemental Fig. 1) was developed with MATLAB (The MathWorks, Natick, MA). Its workflow (Fig. 1) can be summarized as follows:

1. The input Red Green Blue (RGB) images are normalized within the range [0,1] to make the whole set of operations and filtering coherent throughout the whole dataset (Figs. 1a and 1b I). The spectral projection of BV colors in the RGB IHC images covers two of the main colors: red and green. The first step of the algorithm selects the color channels that are utilized to further process the image.
2. Once the red or the green channel is extracted from the full RGB image, a 3 by 3 averaging spatial filter is generated with the MATLAB's built-in function fspecial and it is then applied to the selected color channel to increase the signal to noise ratio. Next, the algorithm suggests an intensity threshold value T to segment the BVs from the background (Figs. 1a and 1b II). The thresholding with the suggested T minimizes the intra-class pixel variance and it is derived from the function graythresh, a MATLAB's implementation of the Otsu's Method.^31^
3. After the thresholding, the binary image is processed to extract all the connected components (Figs. 1a and 1b III). This task is performed by the MATLAB's function bwconncomp, where pixels within an 8x8 spatial neighborhood are considered connected when their edges or corners are adjacent. The connected components are organized into a 1-by-(n) number of detected object array, this set is then reduced by eliminating objects whose areas are smaller than an experimentally derived area threshold. Morphological descriptors, detected with built-in function regionprops, are measured for each of the connected components. The coordinates of all the pixels that belong to the i^th^ connected component are stored in the descriptor PixelList. The number of pixels is used to identify and remove components too small or too large to be blood vessels. This operation has the side effect of removing all the small blood vessels with large lumina. In order to recover them, all round shape objects are identified using the built-in function imfindcircles (Fig. 1b III). The resulting mask is compared to the complement of the original binary image. The intersection highlights all of the hollow regions, which are considered lumina if contained in the circle and larger than 12.5% of the area of the detected object. The components that meet the criteria are reintroduced in the PixelList.
4. Groups of background pixels that are disconnected from the edge of the image are detected and filled (Figs. 1a and 1b IV). The task is performed in two steps. First, the morphological operator bwmorph is used to close the binary images by performing a dilation followed by an erosion. Second, imfill is used to fill the areas within the closed groups of pixels.
5. Connected components are extracted using the binary filter obtained in the previous step and labeled as BVs using labelmatrix (Figs. 1a and 1b V).
6. Regionprops is now used to extract Area, Orientation, Major Axis Length, Minor Axis Length, Eccentricity, Centroid and Perimeter of each labeled element. Finally, the detected vessels are highlighted by overlaying white arrows on top of the starting image (Figs. 1a and 1b VI)

The BVD algorithm is distributed through GitHub: https://github.com/alessandrobruno10/BVD

And MathWorks File Exchange: https://www.mathworks.com/matlabcentral/fileexchange/87422.

### Precision, Recall, F-Measure

In order to evaluate the BVD algorithm performance, the software was quantitatively evaluated using *precision, recall* and *F-measure* parameters.^28,29^ *Precision* is used as a metric for digital image analysis algorithms and measures the positive prediction (see Eq. 2).

2 $${\text{Precision}} = \frac{{{\text{True}}\;{\text{Positive}}}}{{{\text{True}}\;{\text{Positive}} + {\text{False}}\;{\text{Positive}}}}$$

Similarly, *recall* is commonly utilized^28^ to assess the sensitivity of the algorithms. For this specific case, *recall* is defined as follows (see Eq. 3):

3 $${\text{Recall}} = \frac{{{\text{True}}\;{\text{Positive}}}}{{{\text{True}}\;{\text{Positive + False}}\;{\text{Negative}}}}$$

where the *true positive* is the number of correctly detected BVs identified by the algorithm, while *false positive* is the number of artifacts that were incorrectly individuated as BVs, and *false negative* represents the number of BVs that are mistakenly missed by the analysis. A single measure of performance is finally provided by the *F-measure* value, which is defined as the harmonic mean of *precision* and *recall* (see Eq. 4).

4 $${\text{F - Measure}} = {2}\frac{{{\text{Precision}} \cdot {\text{Recall}}}}{{{\text{Precision}} + {\text{Recall}}}}$$

The algorithm performance was tested using a phantom dataset, dataset 1 and 2, respectively. In order to create a set of images that includes a known number of BVs, 10 phantom images have been created by a human operator using Adobe Photoshop 2020 (Adobe Inc.). BVs, with different structures, have been randomly selected from IHC images and added on a black background. The ground truth for the analysis conducted on datasets 1 and 2 was established using the data generated by the human operators.

### Statistical Analysis

Statistical analysis was performed using SigmaPlot (Systat Software Inc.). One-way analysis of variance (ANOVA) and Repeated Measures (RM) ANOVA was utilized for the comparison of multiple samples. Results were considered to be statistically significant when *p* < 0.05. To allow the comparison between the human operator/ImageJ and algorithm analyses in dataset 3, CTGF and pixels were standardized into z-scores. Standardization of CTGF and pixels measurements in each analysis allows comparison between the two scales, even with the use of different assessments. In addition, the agreement between BVD algorithm and human operator analysis (for datasets 1 and 2), and BVD algorithm and ImageJ CTCF quantification (for dataset 3) was studied using the Bland–Altman approach.^11,21^

## Results

### Performance Evaluation of BVD Algorithm on a Phantom Dataset

**Figure 2:**
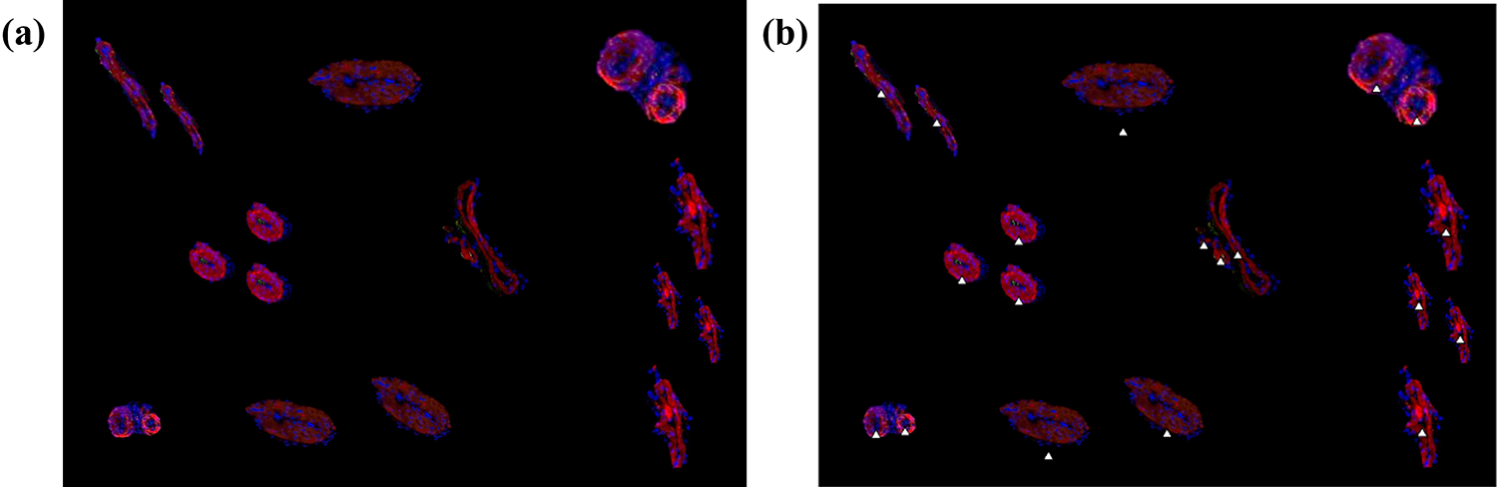
BVD algorithm performance. (a) Phantom image obtained adding stained BVs, with different shapes, on a black background. (b) BVs detected by the algorithm.

The phantom dataset analysis using the BVD algorithm demonstrated that BVs with different shapes and structures were correctly detected within the images. Metrics for the algorithm performance including precision, recall and F-measure were equal to 89%, 95%, 92% respectively (Fig. 2).

### Performance Evaluation of BVD Algorithm on Rat Abdominal Wall Defect Model Dataset

**Figure 3:**
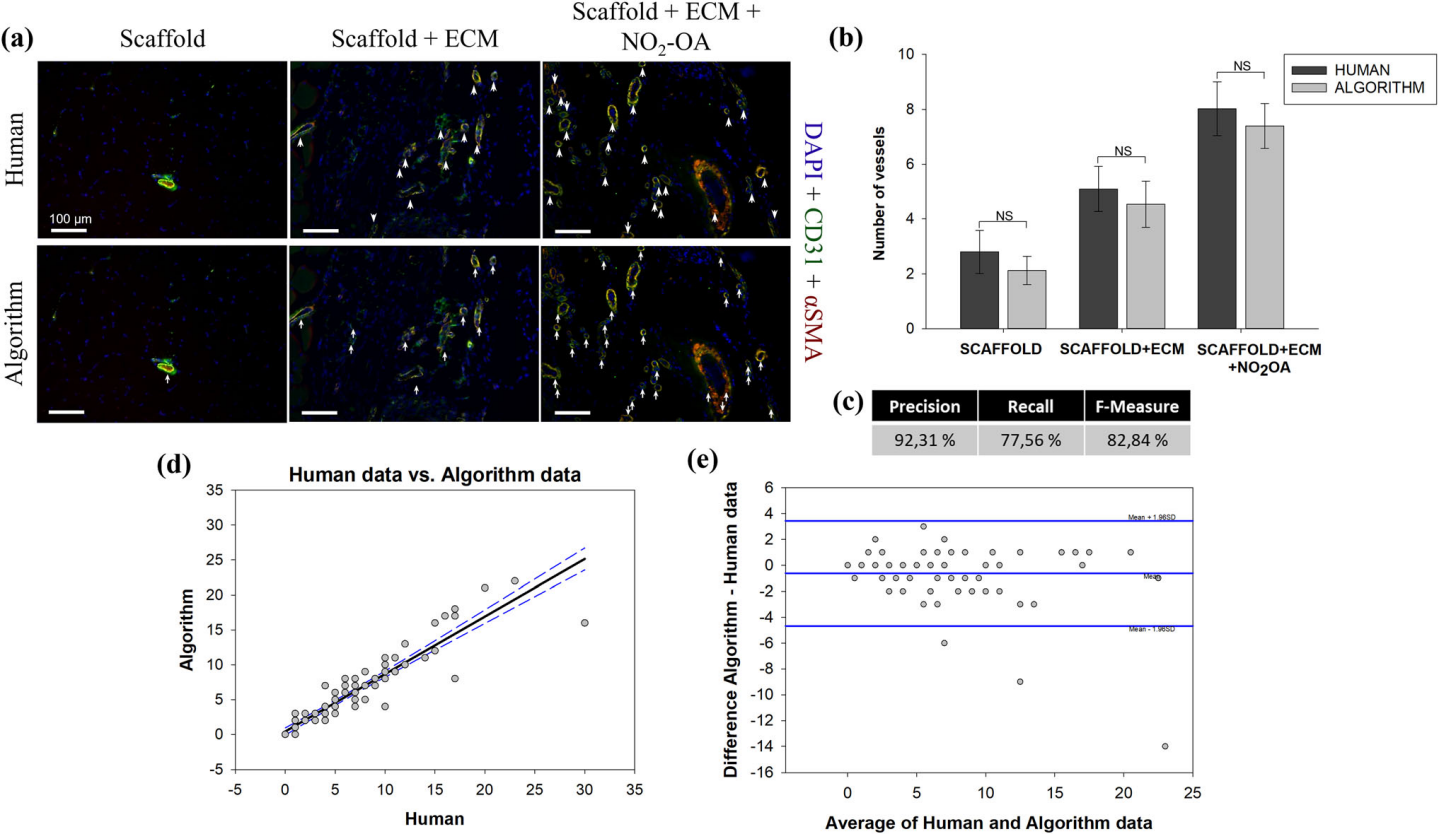
Immunohistochemical staining and analysis for dataset 1: drug-controlled release in rat abdominal wall defect model. (a) BV detection performed by human operators (top row) compared to the detection performed by the BVD algorithm (bottom row), scale bars correspond to 100µm. A total of 96 images were analyzed. For each group, *scaffold*, *scaffold+ECM,* and *scaffold+ECM+NO*_*2*_*-OA*, a representative CD31 + αSMA rat abdominal wall co-stained section is shown. White arrows highlight the identified BVs according to both the human and the algorithm analysis. (b) Bar chart (Mean ± SE) showing the number of vessels detected by the human operator compared to those detected by the algorithm. RM ANOVA statistical test showed no significant differences between the human and the algorithm analysis, indicating a comparable capacity to identify BVs. (c) Algorithm performance is measured with *precision*, *recall,* and *F-measure*. (d) Regression of human vs. algorithm data, with prediction limits. (e) Bland–Altman plot of difference against mean for the BVD algorithm and human data, with mean difference and 95% limits of agreement indicated.

As described within the materials and methods section, CD31 + αSMA co-staining was utilized for BV detection for both the *rat abdominal wall defect* and the *infarction model*. Representative images in Fig. 3a provide an example of the comparative evaluation of the BVD algorithm vs. human detection applied to the *rat abdominal wall defect model* dataset. Three groups of explants with different numbers of BVs were analyzed. The human operator analysis and the BVD algorithm showed a comparable capacity to detect BV locations and number. Quantitative comparison of vessel number/μm^2^ in Fig. 3b showed no statistically significant differences (p < 0.05). Metrics for the algorithm performance, including *precision, recall,* and *F-measure* are reported in Fig. 3c and were equal to 92%, 77%, 82%, respectively. These results were further confirmed by the correlation analysis (*r* = 0.93, Fig. 3d). The Bland–Altman analysis demonstrates agreement between the methodologies with few outliers. The three outliers are due to the algorithm and human variability in detecting the BVs within IHC sections in presence of artifacts. This discrepancy in the analysis is in line with the limitations identified in the BVD algorithm workflow, which will be discussed further in the text. In the Bland–Altman plot, the bias between methods was − 0.615, the limits of agreement for paired observations were − 4.66 to 3.43 (Fig. 3e). These values demonstrated the high performance of the BVD algorithm.

### Performance Evaluation of BVD Algorithm on Rat Infarction Model Dataset

**Figure 4:**
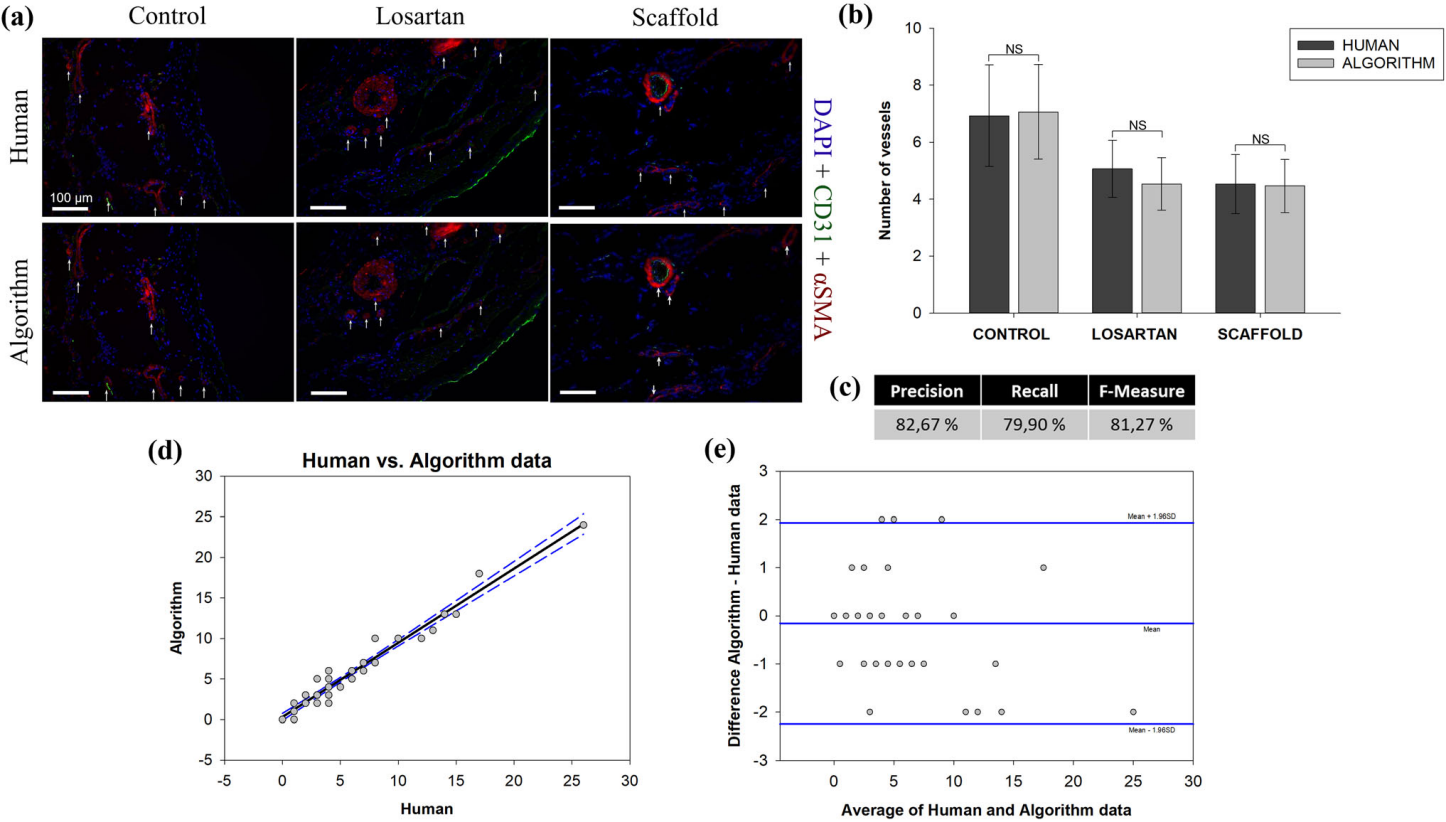
Immunohistochemical staining and analysis for dataset 2: tissue engineered biohybrid scaffold in a rat infarction model. (a) BV detection performed by human operators (top row) compared to the detection performed by the BVD algorithm (bottom row), scale bars correspond to 100µm. A total of 45 images were analyzed. For each group, *control*, *Losartan* and *scaffold*, a representative CD31 + αSMA infarcted rat cardiac tissue co-stained section is shown in panel (a). White arrows highlight the identified BVs in both the human and the algorithm analysis. (b) Bar chart (Mean ± SE) showing the number of vessels detected by the human operator compared to those detected by the algorithm. RM ANOVA statistical test showed no significant differences between the human and the algorithm analysis indicating a comparable capacity to identify BVs. (c) Algorithm performance measured with *precision*, *recall* and *F-measure*. (d) Regression of human vs. algorithm data, with prediction limits. (e) Bland–Altman plot of difference against mean for the BVD algorithm and human data, with mean difference and 95% limits of agreement indicated.

The comparison of the BVD algorithm vs. human results applied to the *host response to biohybrid scaffold* dataset is provided in Fig. 4. More specifically, Fig. 4a shows a qualitative comparison of the capacity to detect number and location of BVs for three different explants groups. Fig. 4b offers and quantitative comparison of the BV quantification that, similarly to the previous case, showed no statistically different values. Finally, *precision, recall* and *F-measure* (Fig. 3c) were equal to 83%, 80%, 81% respectively. The correlation analysis in Fig. 4d showed an *r* = 0.979. In the Bland–Altman plot, the bias between methods was − 0.155, the limits of agreement for paired observations were -2.24 to 1.93 (Fig. 4e). This results corroborated the positive outcomes of the comparative assessment presented for dataset 1.

### Image Artifacts, False Positive and False Negative

**Figure 5:**
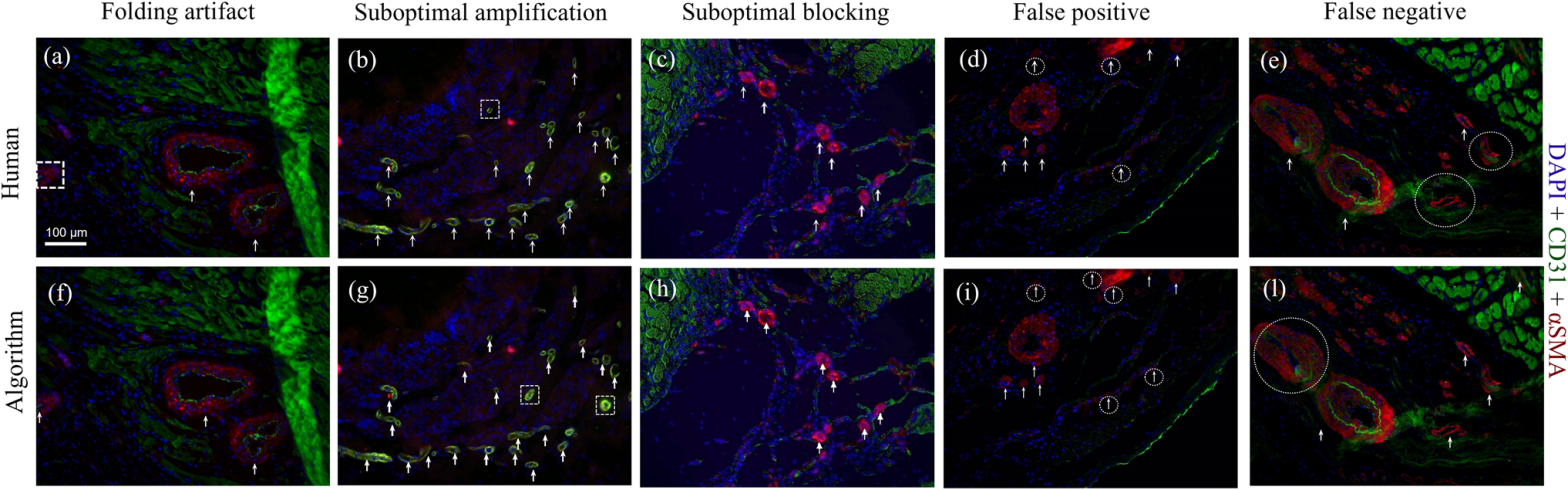
Comparison of human vs. BVD algorithm response to common artifacts. The panel shows different histological sections stained for BV detection (CD31 + αSMA) and analyzed by the human operators and the BVD algorithm, the scale bar is equal to 100µm. Folding (a, f), suboptimal amplification (b, g), suboptimal blocking (c, h) represent common artifacts affecting qualitative and quantitative histological analysis. False positive and negative were also comparatively evaluated. Images a, b, d, e, g, i, and l provide representative examples of this comparison. White dashed circles (false positive), and white dashed boxes (false negative) highlight incorrect or missing BV detections, both from human operator and algorithm analyses. The false negative column (e, l) also provides an example of a suboptimal blocking process (unspecific green staining in the upper right corner). In summary, the response of human operators and BVD algorithm to artifacts was comparable.

Histological sample preparation itself is an important source of variability that, regardless of the methodology being utilized, affects the efficacy and repeatability of the analysis. As discussed in the introduction section, humans are error-prone and often generate false positives and/or false negatives. However, similar errors are also made by algorithms. A comparison of the BVD algorithm vs. human operator in terms of response to image artifacts, false positive and false negative detection is provided in Fig. 5. The three most common causes of image artifacts for the assessment of explanted engineered constructs and biomaterials have been presented, including folding of the histological section, suboptimal amplification, and suboptimal blocking protocol. The algorithm provides an additional level of control to minimize the impact of image artifacts by allowing to manually enhance the thresholding, based on a visual feedback In addition, histogram charts for the diameter and surface distribution of the detected vessels allow the user to identify potential outliers.

### Topological Distribution of Blood Vessel

**Figure 6:**
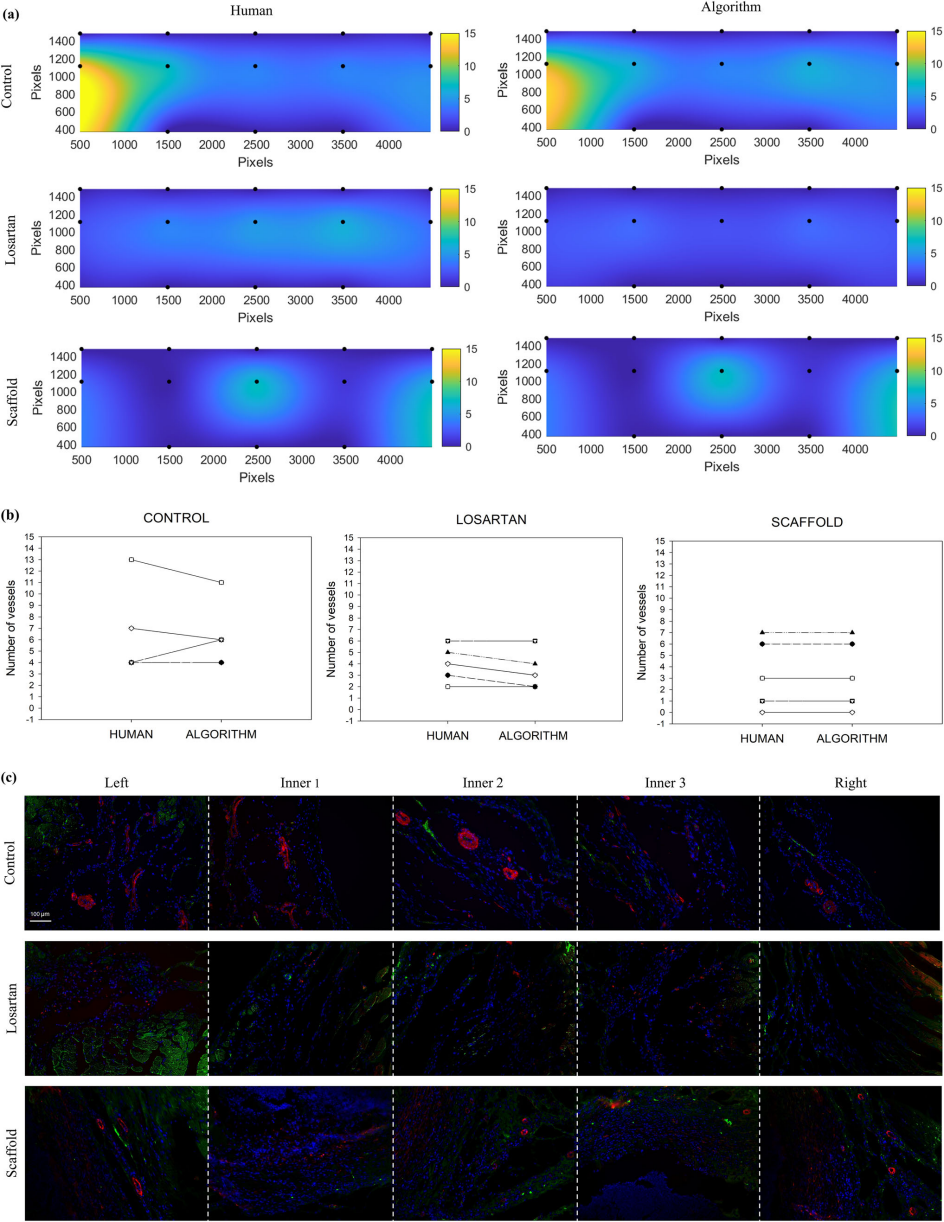
Comparison of human vs. BVD algorithm blood vessel topological analysis. (a) Maps obtained manually by the human operators and automatically by BVD algorithm were comparable in terms of BV number and overall spatial distribution. Samples from dataset 2, "tissue engineered biohybrid scaffold in a rat infarction model", were also analyzed in terms of BV topological distribution. Five adjacent histological sections from each sample were first analyzed and then further processed to obtain a color map of the spatial distribution of the detected BVs. This approach provides additional data as it allows to identify spatial patterns of vessel growth around a region of interest. This information is neglected in the simple quantification of BVs/field of view or the quantification of the fraction of vascular area/field of view. (b) Before and after graphs show differences between human and algorithm BV detection within the samples. (c) Original IHC images mosaic.

In addition to highlighting the locations of the detected BVs (Figs. 3a, 4a), quantifying their total number (Figs. 3b, 4b), their diameter and area distribution (Supplemental Fig. 2), the BVD algorithm can be utilized to obtained color maps that describe the spatial distribution of the BVs inside and around the ROI. An example of such analysis is provided in Fig. 6a and in Supplemental Fig. 2. Given the same number of detected BVs/mm^2^ or BVs/field of view, histological sections can dramatically differ in topological distribution, as is possible to notice by the original images in Fig. 6c. Similarly to the other algorithm's features, a validation was conducted by comparing maps obtained by human operators vs. those obtained by the algorithm. Results presented in Fig. 6a were obtained by processing samples from dataset 2 and showed comparable topological distributions for human and algorithm derived maps. Before and after graphs in Fig. 6b offers a quantitative comparison of the BV quantification that showed no statistically different values.

### Performance Evaluation of BVD Algorithm on Ectopic Kidney Tissue Organogenesis Dataset

**Figure 7:**
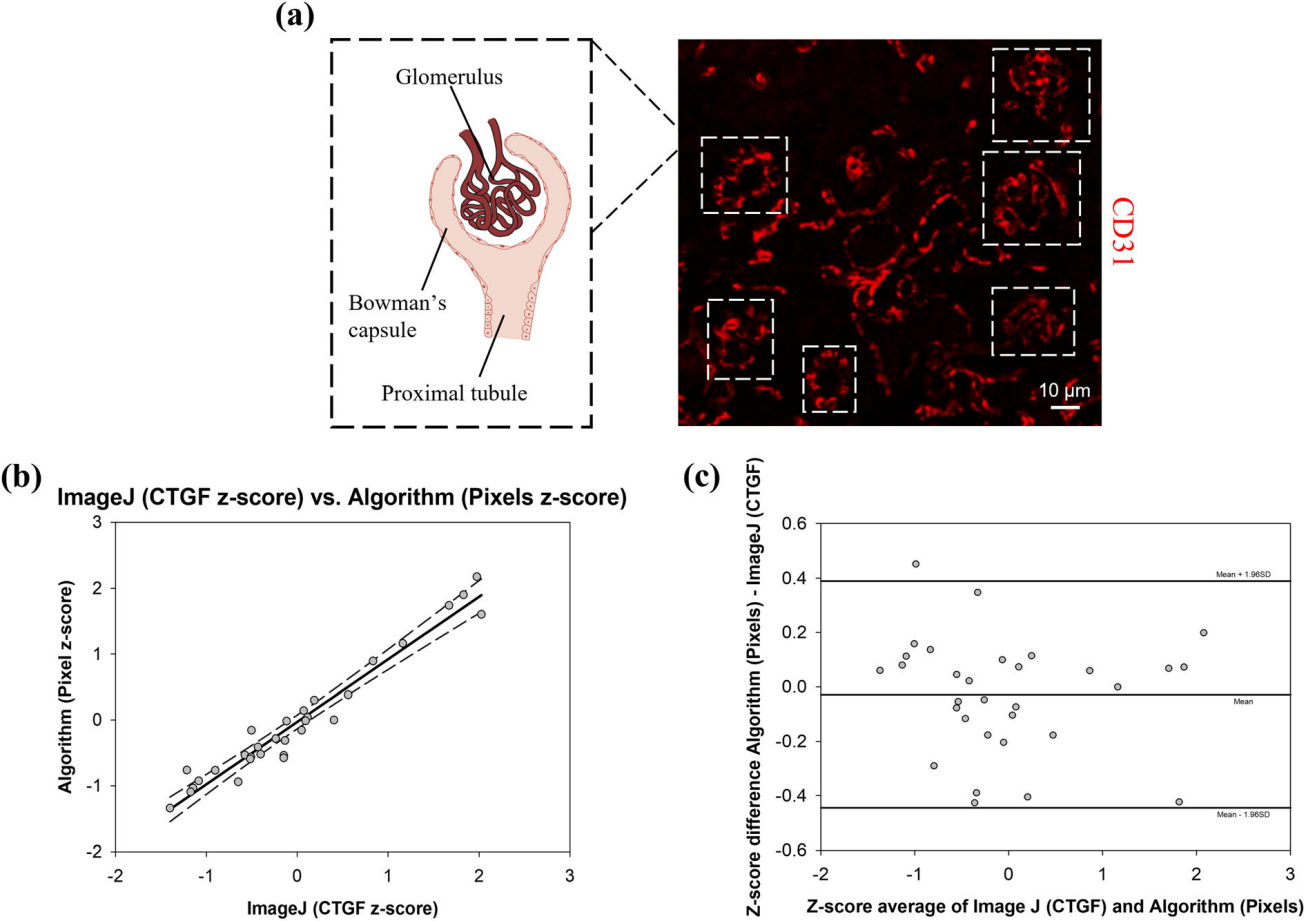
Immunohistochemical staining and analysis for dataset 3: angiogenesis analysis in intraomental kidney graft. (a) Representative immunofluorescence staining for CD31 on a section of intraomental kidney graft and a schematic representation of a glomerulus. Dotted lines highlight six different glomeruli. The glomerulus is a structure specialized for blood filtration and it can be described as a capillary tuft surrounded by the Bowman’s capsule. Scale bar is equivalent to 10µm. (b) A total of 32 glomeruli in five independent IHC sections (≥6/section) were analyzed. Regression of human vs. algorithm z-scores, with prediction limits. (c) Bland–Altman plot of difference against mean for the BVD algorithm and human z-scores, with mean difference and 95% limits of agreement indicated.

The third case that has been considered for the algorithm's validation is conceptually different from the previous two and aims to identify only capillaries within glomeruli of kidney tissue implanted in the omentum.^15^ Capillaries are morphologically characterized by a unique tunica intima and differ from veins, arteries, venules and arterioles that are composed by three layers. The tunica intima consists of a single layer of connected ECs that are positive to the CD31 marker. Therefore, CD31 was the only staining adopted in dataset 3. This specific example was provided to show how the BVD algorithm can be applied in the context of ectopic regeneration and how it can be utilized in the event of an analysis based on an individual staining. As described in the methods section, CTGF was calculated by a human operator, using ImageJ, evaluating the number of red pixels within the ROI. Similarly to the analysis conducted using ImageJ, the ROI was manually identified by the operator around the Bowman’s capsule containing the glomerular capillaries (Fig. 7a). Bland and Altman analysis was used in order to assess the agreement between the z-scores of the two methodologies. The correlation analysis for five independent image acquisitions was performed and it is provided in Fig. 7b, r was equal to 0.97. In the Bland–Altman plot, the bias between methods was 0.27, the limits of agreement for paired observations were − 0.44 to 0.38 (Fig. 7c).

## Discussion

Both research and clinical practice share the urgent need for advancing experimental and digital processing methods to visualize and quantify targeted biomolecules. The notion of quantitative analysis and proper synthesis between software engineering and histopathology is applicable to a broad spectrum of applications. In this regard, numerous factors are recognized as a source of variability, including the staining procedure and the subjectivity introduced by the human operator. As facilitated access to large datasets increasingly becomes a determinant element, reducing digital processing time has become more of a necessity rather than a design requirement.

In this study, an automated, ready-to-use and user-friendly methodology to quantitatively characterize BVs in immunohistochemical sections was presented. The accuracy of the results was assessed using a phantom dataset of images and by the direct comparison with results obtained by human operators analysis using state-of-the-art methodologies. Three different datasets have been utilized for the algorithm evaluation, including: a drug-controlled release study^7^ (Fig. 2), the host response to the implantation of a biohybrid scaffold^35^ (Figs. 3 and 5), and an example of ectopic kidney organogenesis induced by transplantation of kidney rudiments in the omentum^15^ (Fig. 7). Algorithm's performance was further quantified on a phantom dataset with well accepted metrics such as *precision, recall, F-measure* (Fig. 6). Response to common artifacts was also presented (Fig. 4).

Results in Figs. 2, 3 showed the ability of the BVD algorithm to quantify the number of BVs that were statistically equivalent to those measured by a human operator. Unlike human operator-based analysis, the information provided by BVD was comprehensive and included a set of data that is generally neglected in histological analysis. For instance, in addition to the location and number of BVs, area, and diameter were calculated and presented as histogram charts (Supplemental Fig. 2). The number of vessels can be provided as number/mm^2^ rather than being offered as number of BVs/fields of view. The algorithm efficacy was documented (Figs. 2c and 3c) with *precision* (>82%)*, recall* (>77%)*, F-measure* (>81%).

A trained human operator may spend about two minutes/image identifying and counting BV on a histological section and saving the data. Considering the size of a standard pre-clinical model with 10 images/explants, 5 explants/group, and 3 groups, the processing time for a manual operator is equivalent to a minimum time commitment of 300 minutes. This estimate not only represents a theoretical lower limit, but also does not include other forms of analysis or post-processing such as the creation of arrays with BV areas, diameter or centroid locations. In contrast, the BVD algorithm eliminates human subjectivity, does not require trained operators, and requires less than 20 seconds to complete the analysis on a conventional laptop. This is equivalent to a reduction in the processing time of a factor of six. A GUI was also provided and offers additional choices for the analysis. For instance, single images can be analyzed in any of the RGB channels. Detection can be optimized for the specific tissue source and staining by customizable gray-level thresholding. Sets of congruent images from the same dataset can be used to produce an interpolated topological map showing the spatial distribution of its BVs (Fig. 5). The integration of quantitative histology in clinical and basic science research is at its early stage.

While several techniques have been previously developed to analyze vasculature and angiogenesis, the majority of them are not-open source, not automatic, require manual interaction of an expert operator or need a training phase. The state-of-the-art for open-source quantitative BV analysis consists of only one method: CAncer IMage ANalysis (CAIMAN).^31^ CAIMAN is an algorithm repository designed to analyze images for cancer research. Only semi-quantitative assessment was provided by comparing the automatic segmentation with hand delineation of BV edges on human colorectal carcinomas in mice models and mouse fibrosarcoma. Yet, the algorithm provides information such as vascular area, eccentricity, and roundness.^31,32^ On the other hand, CAIMAN is able to process IHC images stained with chromogenic dyes and acquired by a bright-field microscope. Although the CAIMAN’s official repository is no longer maintained, the source codes are available on the developer’s GitHub.

The original scripts are not suitable for the analysis of IF images and require the use of MATLAB’s interface. However, this seminal study has the merit to highlight the important gap in knowledge created by the different methodological approaches adopted in histopathology and engineering.

The current study introduces an algorithm capable of performing quantitative analysis of BV morphology regardless of tissue source, scale, or presence of implanted biomaterials. The algorithm is provided as a stand-alone application that does not require an understanding of the programming language, runs off-line on a personal computer, and does not require specific software to be installed. BV number, the conventional parameter in the study of angiogenesis, is augmented by additional morphometrical data such as BV locations, diameters, and areas. This information can be beneficial for numerical modeling, and it is increasingly gaining interest in computational methods to predict the evolution of the inflammatory response.^43^ The quantitative analysis also allows, for the first time, to characterize the topological distribution of the BV around the ROI. This long-time neglected feature is a valuable tool to distinguish different vascular patterning that, while similar in vessel density, may differ in terms of topology.

A number of limitations can be identified for this study. First, the algorithm was developed to process immunofluorescent images only, this category benefits from the suppression of the background performed by the staining procedure. While histological images of BVs processed by other staining such as hematoxylin and eosin or Masson's trichrome are morphologically similar to those obtained by IHC staining, this class of images will be hardly analyzed by the BVD algorithm. Second, the morphological operators implemented in our method were prevalently calibrated to detect round-shape objects. While longitudinal sections are still detected, the accuracy of the method in such cases is sub-optimal. Third, the BVD algorithm has a limited ability to separate neighboring BV when they are too close. We plan to address this issue in future releases of the application. Fourth, the isolation of the ROI within an image is still manually identified by the human operator before the analysis. Last, deep learning based approaches such as fully convolutional networks for semantic segmentation^10^ could be utilized and could potentially provide competitive advantages compared with the current implementation. While promising, deep learning for the analysis of vascularization is currently at its initial stage and would also come with the common caveat of defining effective procedures and suitable dataset for the training.

BVD algorithm is shown to be effective in automatically identifying and quantifying vascular patterns in immunohistochemical sections. The BVD is provided as an open-source application that allows a comprehensive morphometrical and topological characterization of BVs. This method can facilitate histopathological assessment in a broad spectrum of pre-clinical scenarios where unbiased and faster quantification is relevant.

## Supplementary Information

Below is the link to the electronic supplementary material.

Supplementary file1 (DOCX 2825 kb)
