# Supplementary material for: Blood Vessel Detection Algorithm for Tissue Engineering and Quantitative Histology
Source: Ann Biomed Eng. 2022 Feb 16;50(4):387–400. doi: 10.1007/s10439-022-02923-2 (PMC8917109; doi:10.1007/s10439-022-02923-2)
Supplement: Supplementary file 1 — Supplementary file1 (DOCX 2825 kb) [file 10439_2022_2923_MOESM1_ESM.docx]

**SUPPLEMENTARY MATERIAL**

***Algorithm Graphical User Interface (GUI)***

***
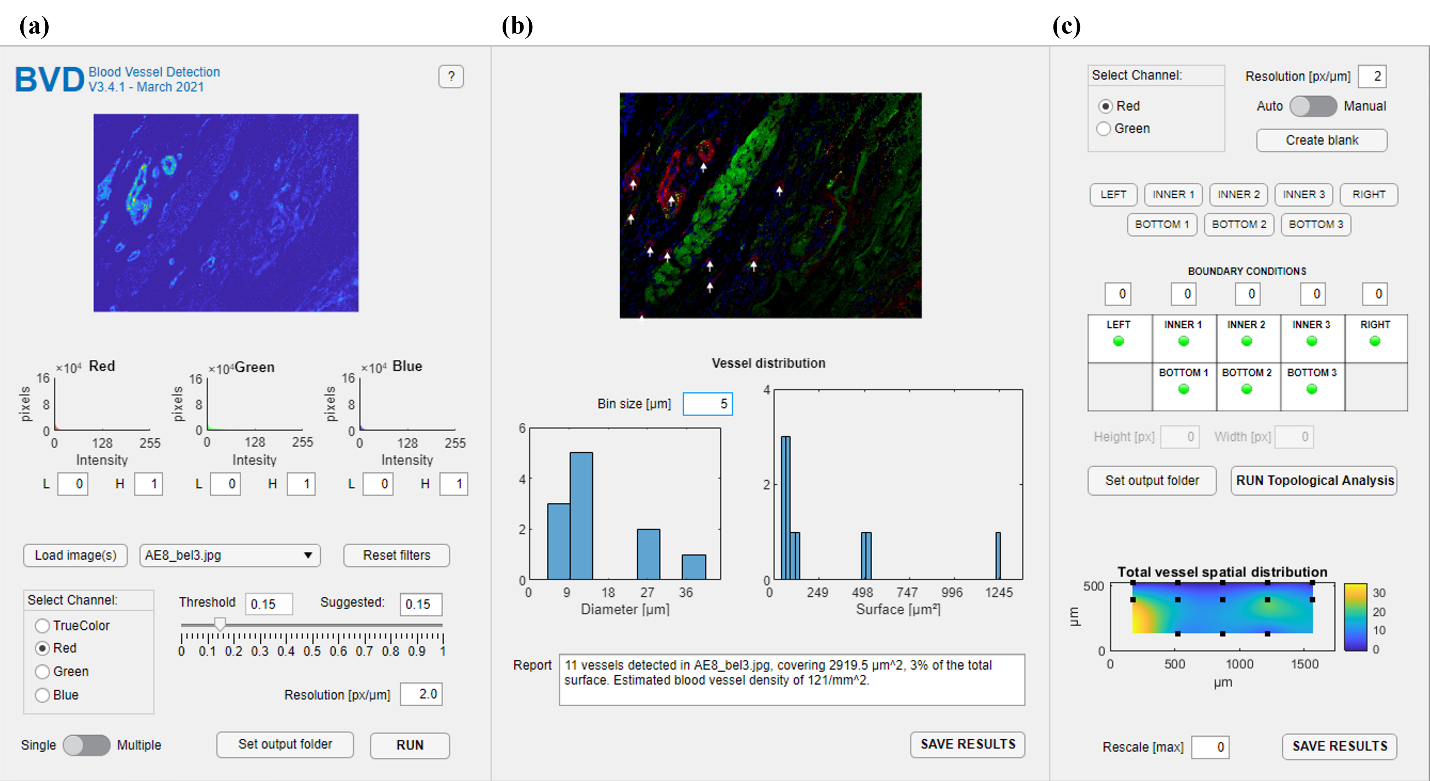
***

**Supplemental figure 1. Algorithm Graphical User Interface (GUI).** Figure shows a representative image of the BVD algorithm GUI. The GUI is divided in three main panels. The input panel (a) allows to upload and visualize the images, and allows to set further parameters for the analysis (e.g., filters, channels, threshold, resolution). The output panel (b) provides a summary of the analysis. Results include the original image with arrows highlighting the detected BVs, the vascular areas and diameter histograms and a report window. The “Report” box offers a brief description of the analysis that specifies the total number of detected BVs, the total area covered by BVs, expressed in µm^2^ and fraction of the entire image, raw data are saved as a TXT file. The third panel (c) was designed for the topological analysis. This section supports two different type modalities: “*Automatic*” or “*Manual*”. Using the “*Automatic*” mode a combination of up to eight contiguous images can be used to automatically create an interpolated composite color map of the BV spatial distribution. In the “*Manual*” mode, the number of BVs, obtained by previous analysis of each specific image or arbitrarily inputted by the user, will be provided interactively by the user to obtain the vessel spatial distribution. Warmer colors indicate a higher number of BVs as specified by the color legend positioned at the right hand of the map, this particular example provides a 0-30 BV range. The black dots within the map show the geometrical center of each image. Visual results can be saved and exported in TIFF format.

The Graphical User Interface (GUI), was developed in MATLAB App Designer and it is shown in Figure 2. BVD is a fully automated method that extracts from histological images the number of BVs, locates their centroids, and computes the vascular fraction of the image. Furthermore, in addition to the detection of BVs and to the quantification of a number of morphological descriptors, when adjacent histological images are provided, users can perform the topological analysis of the vessel spatial distribution in proximity to a ROI. This feature is particularly relevant in the context of biomaterials and tissue engineering when assessing the host response to a medical device or an engineered construct. The GUI is divided into three panels. The input panel (Figure 2a) is used to input key parameters and initiate the image pre-processing. A link to the user manual is provided in the top right corner. The GUI input panel offers a set of commands to load and display the images, modify the contrast of each RGB channel, select the channel on which the analysis will be performed, input the resolution [px/μm], set how to save the output data, and start the analysis. Once inputs are provided, the software automatically computes the thresholding value as described in the previous methods section, yet users can re-adjust to a different value using the slider bar. The input panel also contains a switch that allows to run the analysis on a set of images using the automatic thresholding value. This feature is meant to allow for the processing of large datasets and is based on the assumption that the targeted color channel remained unmodified for all of the images within the set. The middle panel (Figure 2b) visualizes the results of the analysis. The main figure on the top of the panel shows the position of the identified BVs that are highlighted with white arrows. The two charts on the bottom of the middle panel provide the histogram distribution of the detected BV diameters and areas. Binning resolution can be adjusted interactively. A written report is also provided including the number of detected vessels, their cumulative surface, in µm^2^ or as percentage of the entire image surface. The written report, histograms raw data, BV centroid positions, and output TIFF images can be saved and exported. The third panel of the GUI (Figure 2c) is designed for assisting the topological analysis. Up to 8 adjacent images surrounding a desired ROI can be utilized for the analysis according to the grid shown in the panel. The imaging of these eight regions, while not a mandatory requirement for the algorithm, allows for a precise characterization of a ROI^8, 9^. These 8 areas are labeled as: left, right (around the ROI), inner 1, inner 2, inner 3 (the ROI), bottom 1, bottom 2, bottom 3 (below the ROI). This topological analysis is useful for a number of common *in vivo* models assessing engineered devices and biomaterials, examples include epicardial placement^9^ of cardiac patch or partial and full abdominal wall replacement^2, 8, 44^. If the “*Auto*” mode is selected, the user will use the set of 8 buttons that corresponds to the 8 areas of the grid to load the respective images, and the algorithm will automatically run the BV detection analysis as previously explained. The “*Auto*” mode automatically calculates the size of each image, finds its center, and spatially positions the adjacent images accordingly so that one unique map is obtained. The output image at the bottom of the panel shows a composite map, obtained interpolating the values of each analysis conducted in the evaluated areas. This color map provides quantitative and continuous spatial distribution of BVs around the ROI. If needed, users can readjust the range of the colormap using the "*Rescale"* input field. This function can be utilized to compare results between different sets of images that may be characterized by a different maximum number of detected BVs. As for the previous panels, results, including raw and visual data, can be saved and exported. Additionally, a *“Manual”* mode was included and allows for simple visualization or for comparing the "Auto" detection with results obtained with other methodologies. If this mode is selected, the interface will change and allow to manually input the number of vessels for each of the areas. In the *“Manual”* mode, the size of the output map depends on the *Height* and *Width* values specified by the user and it is assumed to be the same for all the areas of the grid (Supplemental Figure 1).

**Topological analysis**

**Supplemental figure 2. Comparison of human vs. BVD algorithm blood vessel topological analysis.** Samples from each group of dataset 2, tissue engineered biohybrid scaffold in a rat infarction model, were analyzed and comparatively evaluated as described in Figure 6. The GUI allows for two different modalities to execute the algorithm: I) adjacent images can be either uploaded as individual set, analyzed and processed automatically (*Algorithm*); II) the GUI can be utilized to build the color map by sequentially inputting the detected BV number that was previously calculated with a method of choice (*Manual input*).

**Blood vessel morphological analysis**


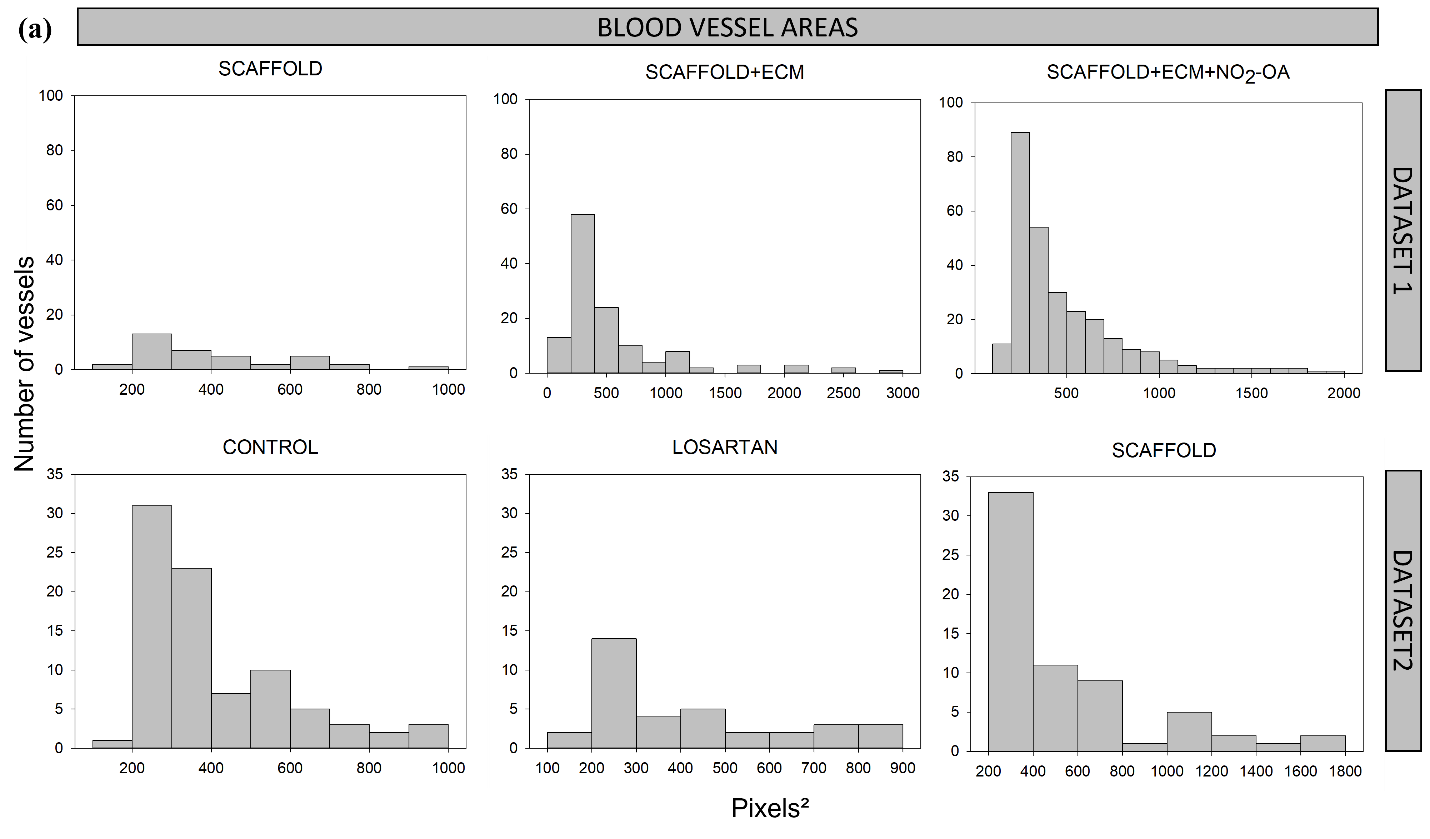


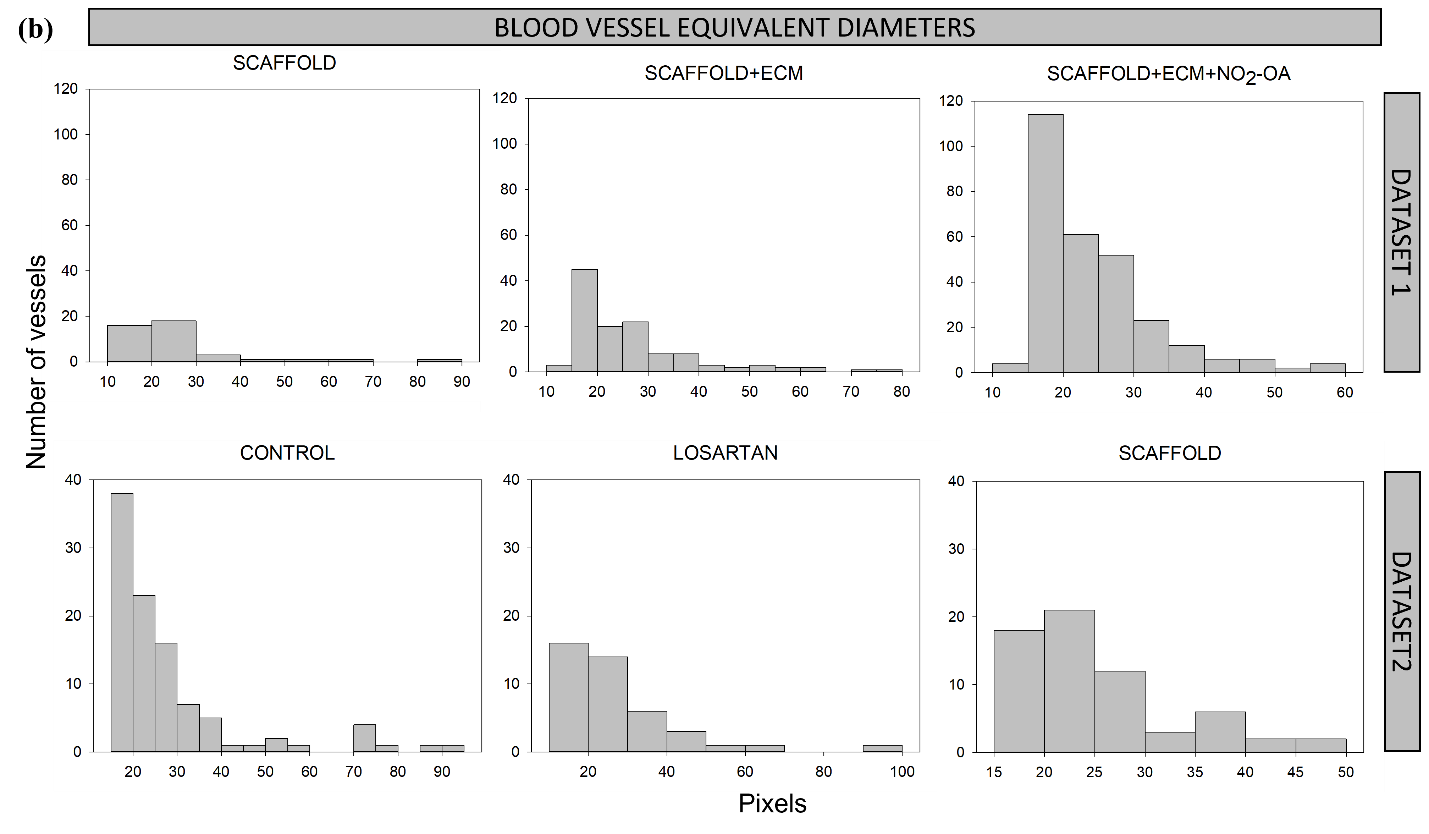


**
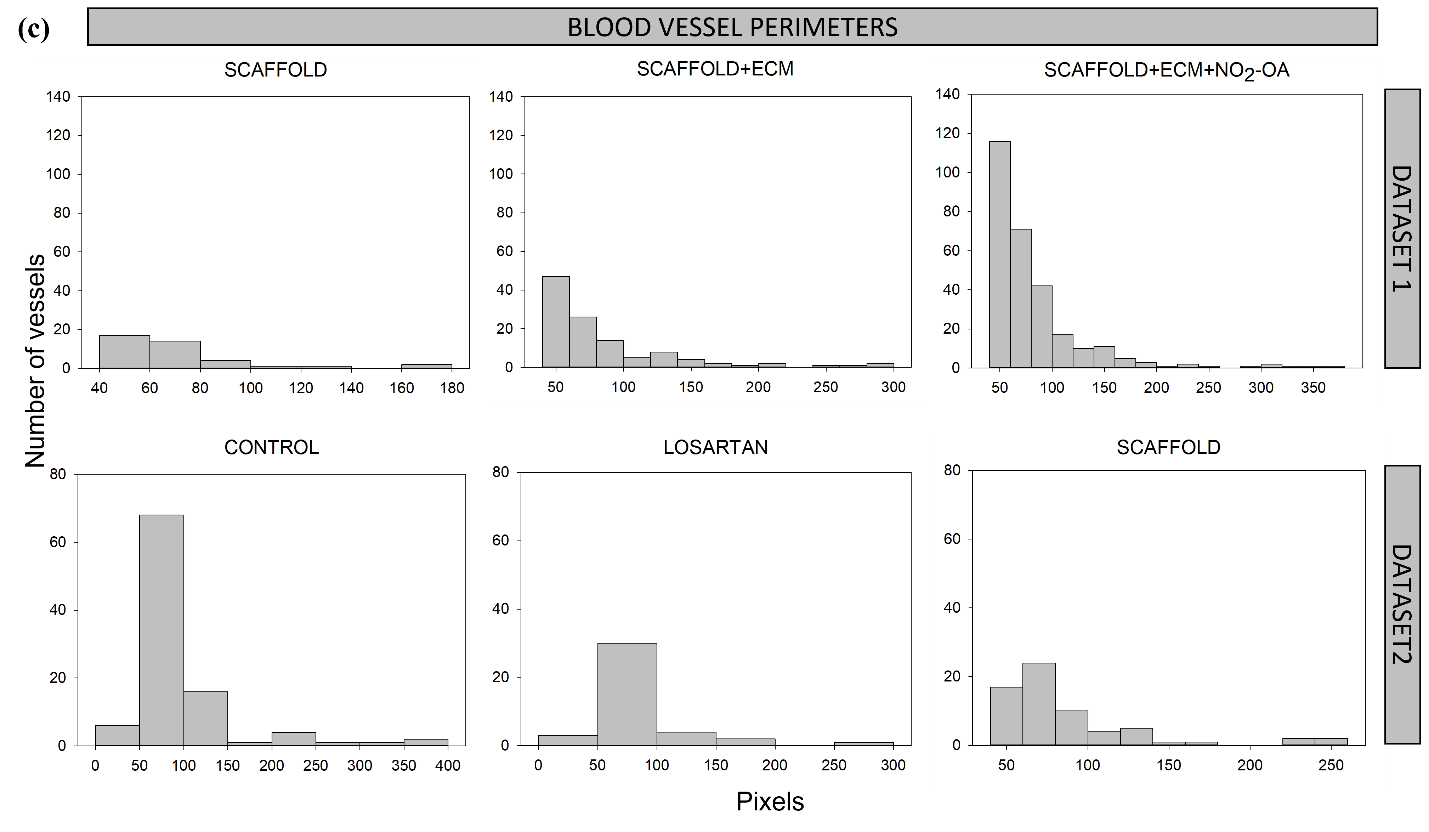
**

**
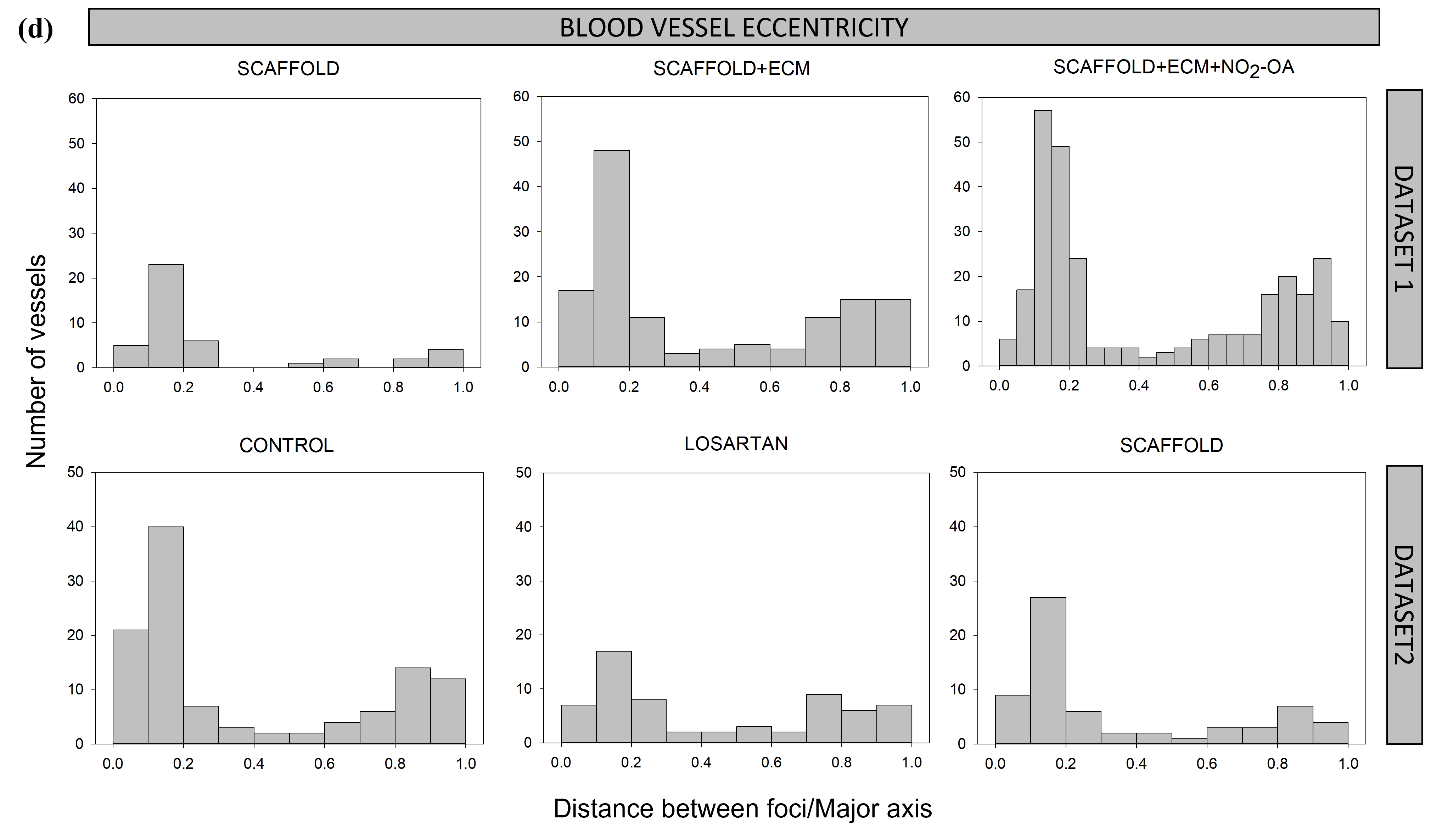
 Supplemental figure 3. Blood vessel morphological analysis.** Histograms showing the distribution of blood vessel areas (a), equivalent diameters (b), perimeters (c), and eccentricity (d) within the groups in datasets 1 and 2.
